# Using coherent X-rays to follow dynamics in amorphous ices†

**DOI:** 10.1039/d2ea00052k

**Published:** 2022-09-13

**Authors:** Marjorie Ladd-Parada, Hailong Li, Aigerim Karina, Kyung Hwan Kim, Fivos Perakis, Mario Reiser, Francesco Dallari, Nele Striker, Michael Sprung, Fabian Westermeier, Gerhard Grübel, Anders Nilsson, Felix Lehmkühler, Katrin Amann-Winkel

**Affiliations:** Department of Physics, Stockholm University Roslagstullsbacken 21 10691 Stockholm Sweden amannk@mpip-mainz.mpg.de; Max-Planck-Institute for Polymer Research Ackermannweg 10 55128 Mainz Germany; Department of Chemistry POSTECH Pohang 37673 Republic of Korea; Deutsches Elektronen-Synchrotron DESY Notkestr. 85 22607 Hamburg Germany; Hamburg Centre for Ultrafast Imaging Luruper Chaussee 149 22761 Hamburg Germany; Institute of Physics, Johannes Gutenberg University Mainz Staudingerweg 7 55128 Mainz Germany

## Abstract

Amorphous solid water plays an important role in our overall understanding of water's phase diagram. X-ray scattering is an important tool for characterising the different states of water, and modern storage ring and XFEL facilities have opened up new pathways to simultaneously study structure and dynamics. Here, X-ray photon correlation spectroscopy (XPCS) was used to study the dynamics of high-density amorphous (HDA) ice upon heating. We follow the structural transition from HDA to low-density amorphous (LDA) ice, by using wide-angle X-ray scattering (WAXS), for different heating rates. We used a new type of sample preparation, which allowed us to study μm-sized ice layers rather than powdered bulk samples. The study focuses on the non-equilibrium dynamics during fast heating, spontaneous transformation and crystallization. Performing the XPCS study at ultra-small angle (USAXS) geometry allows us to characterize the transition dynamics at length scales ranging from 60 nm–800 nm. For the HDA-LDA transition we observe a clear separation in three dynamical regimes, which show different dynamical crossovers at different length scales. The crystallization from LDA, instead, is observed to appear homogenously throughout the studied length scales.

## Abstract 2

Amorphous ice plays an important role in our overall understanding of water's phase diagram.

## Graphical abstract

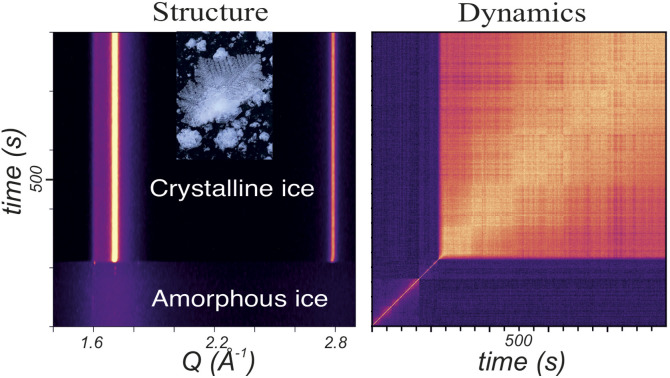

**Environmental significance**

Unravelling the fundamental properties of water and water's phase diagram is highly relevant for our understanding of water in our environment. Moreover, amorphous ice was predicted to occur under summer mesospheric conditions, prior to crystallising into hexagonal ice. Our studies give insight into the structural and dynamical properties of different amorphous ices and their crystallization.

## 1. Introduction

Water's phase diagram exhibits an extraordinarily large number of different solid states, and while some have been found or predicted to occur in nature, others only exist in a laboratory environment. Amorphous ice is assumed to be the predominant state of solid water in space.^1–3^ It forms on cold dust grains in interstellar clouds or comets,^4,5^ and is predicted to participate in many chemical reactions in outer space due to its porous morphology. The water molecules in amorphous ice appear to be disordered and not arranged in a crystal lattice.^6^ On Earth, however, the most stable solid phase is hexagonal crystalline ice (I_h_).^7^ Nevertheless, it has been proposed that amorphous ice can nucleate under mesospheric conditions and eventually crystallize upon heating.^8,9^

Whilst different amorphous ices have been identified, largely due to the different methods of preparation in the laboratory,^6^ they can usually be divided into high- and low-density amorphous ice (HDA and LDA).^10^ These two solid states are proposed to be related to the high- and low-density liquid states of water^11^ (HDL and LDL). In this context, the origin of water's anomalous properties can be explained within the liquid–liquid critical point hypothesis by the existence of two liquid states of water.^12,13^ The two states can only separate below this critical point, hence at low temperatures and slightly elevated pressure. However, other scenarios have been proposed which do not include such a second critical point.^14–16^

Different experimental approaches have been used to investigate the properties and structural changes of water at different temperatures and pressures to understand the relationship between supercooled water and amorphous ices.^11,17–21^ Amongst them we find studies on the thermodynamic properties of water at supercooled conditions^22^ and the structure of the metastable liquid states.^23^ Investigating the molecular water dynamics upon heating amorphous ices might be the key for our understanding on how amorphous ices are related to their liquid counterparts.^11,24^ While most of the recent experimental data converge to a picture of water consisting of two-liquids,^18,24^ a few questions remain open as some experiments are not consistent with this scenario.^25–27^

Unravelling the mechanism of water's phase transitions and the interconnection between amorphous ices and liquid water plays an important role in our overall understanding of water in environmental processes. Here, we combine wide-angle X-ray scattering (WAXS), ultra-small-angle X-ray scattering (USAXS) and X-ray photon-correlation spectroscopy (XPCS) to probe both the structural and dynamical properties of amorphous ices at various conditions, in particular during phase transition and crystallization.

## 2. Methods

### 2.1 Sample preparation

**Fig. 1:**
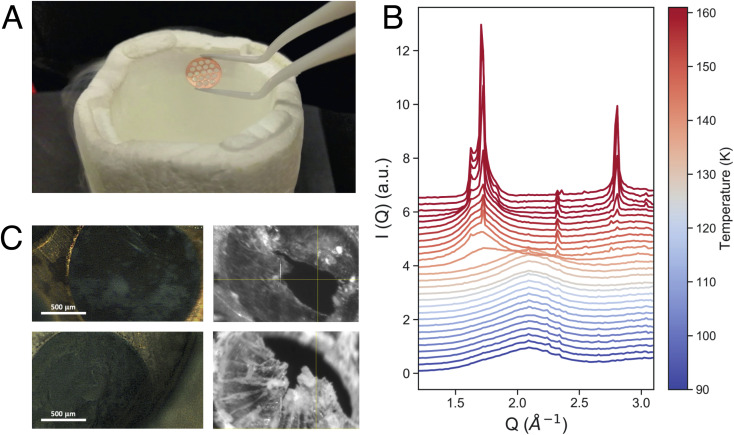
A). Picture of the copper grid, inside of which the ice samples are formed. (B) Azimuthally integrated intensity in the WAXS range of an eHDA sample heated from 90 K to 160 K at 10 K min^−1^. (C) Four microscopy pictures of different amorphous ice samples.

Equilibrated high-density amorphous ice (eHDA) was prepared by using a piston cylinder setup and a material testing machine (Zwick, Z100 TN).^23,28,29^ In brief, a 0.1 mm-thick Cu-disk with 1.5 mm hole-structure was filled with ultrapure deionized water and precooled to form crystalline ice (Fig. 1A). Note that preparing the sample inside the Cu-disk is distinctly different to usually prepared bulk samples. After loading the sample into the piston setup, it was pressurized to 1.6 GPa at 100 K, forming unannealed high-density amorphous ice (uHDA). The sample was then decompressed to 1.1 GPa and annealed at 160 K, and subsequently decompressed further to 0.08 GPa at around 140 K, allowing the transformation into eHDA. The sample was quenched to 80 K, extracted at ambient pressure and stored in liquid nitrogen until use.

### 2.2 X-ray scattering

#### 2.2.1 Wide-angle X-ray scattering (WAXS)

The fast WAXS measurements were performed at the XSS-FXS beamline of PAL-XFEL, Pohang, South Korea at a photon energy of 9.7 keV with a focal size of 19 μm × 32 μm. The scattering patterns were taken in the WAXS regime covering a *Q*-range of 0.1–3.2 Å.^−1^

#### 2.2.2. Simultaneous WAXS and X-ray photon correlation spectroscopy (XPCS)

The combined WAXS and XPCS experiments were performed at beamline P10 at the Deutsches Elektronen-Synchrotron (DESY), Hamburg, Germany. The XPCS experiments were conducted in ultra-small-angle (USAXS) geometry, while the structure was monitored simultaneously by WAXS. We used a photon energy of 8.4 keV with an unfocused X-ray beam of 100 μm × 100 μm. The XPCS patterns were recorded with an Eiger X4M detector, located 21.2 m downstream from the sample. For each dataset a total of 1000 frames were taken with an exposure time of 1 s per frame. The WAXS patterns were recorded simultaneously with an Eiger 500K detector located 147 mm from the sample.

The dynamics are calculated using the temporal intensity autocorrelation function:^30–33^1
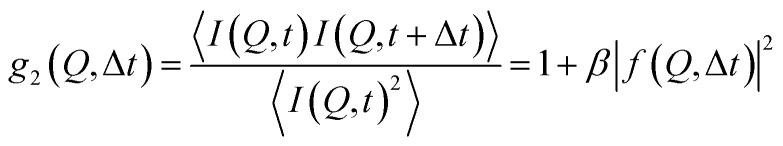
where *I* (*Q*, *t*) is the intensity at the modulus of the momentum transfer *Q* = 4πsin(2*θ*/2)/*λ*, Δ*t* denotes the correlation time delay, and the brackets 〈…〉 indicate averaging over all detector pixels within the same *Q* interval as well as all frames at different times *t*. *β* is the speckle contrast and *f* (*Q*, Δ*t*) is the intermediate scattering function that contains information about the sample dynamics. Typically, it can be modeled in a stretched exponential form:^24,33–35^

2 *f*(*Q*, Δ*t*) = exp{−[*Γ*(*Q*)Δ*t*]^*γ*^}

where *Γ*(*Q*) is the *Q*-dependent relaxation rate (*Γ* = 1/*τ* where *τ* is the relaxation time) and *γ* is the Kohlrausch–Williams–Watts (KWW) exponent. Thus, for a simple system the dynamics can be described by:

3 *g*_2_(*Q*, Δ*t*) = 1 + *β* exp{−2[*Γ*(*Q*)Δ*t*]^*γ*^}.

Brownian diffusive processes are characterized by *γ* = 1, super-diffusive motions typically show compressed exponentials with *γ* > 1 (ballistic *γ* = 2), and glassy motions often result in stretched correlation functions with *γ* < 1. If a diffusive motion is present, *Γ*(*Q*) has a linear relationship with *Q*^2^: *Γ*(*Q*) = *D*_0_*Q*^2^, where *D*_0_ is the diffusion coefficient.^30,33^

### 2.3 Sample environment

X-ray scattering experiments were performed using a JANIS liquid nitrogen cryostat with a customized sample holder. For the fast WAXS experiments at PAL-XFEL, a cryostat model ST400 was used, while for the XPCS measurements at DESY model VPF-100 was implemented to the beamline. In both cases, differently from previous studies,^24^ the sample was mounted as a free-standing ice-sheet (on average 60 μm thick) within a vacuum chamber (10^−5^ mbar) directly connected to the X-ray path, to avoid any additional scattering from protecting window material. Fig. 1C shows four microscope images of such a sample. Temperature control was achieved by using a Si-diode mounted at the bottom of the cold-finger, a resistive heater cartridge (50 Ω) placed close to the diode, and a Lakeshore temperature controller. All temperatures stated in the manuscript are the measured cryostat-temperatures at the bottom of the cold finger. We assume a small offset between the given cryostat-temperature and the sample temperature itself, due to varying thermal contact when loading the sample under cryogenic conditions. This can vary from loading-to-loading and the sample temperature was detected to be up to 5 K higher than the given cryostat-temperature.

## 3. Results and discussion

### 3.1 The high-to low-density transformation

#### 3.1.1 Structural transformation during fast heating

X-ray studies on the structural transition from high- to low-density amorphous ices have been performed on bulk powder samples, usually prepared as unannealed HDA (uHDA) or very-high-density amorphous (VHDA) ices.^36–39^ The heating rates in those studies are around 100-times slower (0.05 – 0.5 K min^−1^). Here we use short X-ray pulses from PAL-XFEL to follow the transition in the novel thin sample geometry, with single shot measurements using a relatively large heating rate of 10 K min^−1^. Similar heating rates have been previously used in calorimetry,^28^ revealing an exothermic HDA→ LDA transition preceded by an endothermic feature that is discussed to be related to the glass transition in eHDA.^40^

Fig. 1B shows the measured intensity *I*(*Q*) in the momentum transfer range of 1.3 Å^−1^ < *Q* < 3.2 Å^−1^. At 90 K, the starting point of the fast heating, we can observe the characteristic first diffraction maximum of HDA at *Q* = 2.1 Å^−1^. During heating, we observe the appearance of a shoulder at *Q* = 1.7 Å^−1^ and a sharp transition to LDA at 137 K (Fig. 1B). The overall behavior of the eHDA → LDA transition is in agreement with previous X-ray measurements by Mariedahl *et al.*^41^ on bulk powder samples, exhibiting phase coexistence by a bimodal development in *S*(*Q*) rather than a continuous shift in the first diffraction maximum, as observed in uHDA.^36–39,41^ The transition temperature *T*_*t*_ is identical to calorimetric data using the same heating rate of 10 K min^−1^,^40^ while Mariedahl^41^ observes *T*_*t*_ to be at around 124 K (0.5 K min^−1^) and 127 (4 K min^−1^). The HDA to LDA transformation was followed by crystallization to a stacking disordered crystalline ice as expected from both experiments and simulations of the early stages of crystallisation at ambient pressure.^42,43^

#### 3.1.2 Structural transformation during slow heating, accompanied by dynamical investigations

XPCS is used to investigate the molecular dynamics at different selected temperatures throughout the transition from HDA to LDA. The sample was annealed for 20 min at each temperature step to allow for thermal equilibration. Then a scattering pattern series over 1000 s with 1000 images (1 s per image) was taken. We correlated the measured intensity over time to obtain information about the underlying motions. This was done at momentum transfer of 0.0008 Å^−1^ < *Q* < 0.018 Å^−1^ in the ultra-small X-ray scattering regime (USAXS), allowing us to follow structural changes on the nm-length scale.

**Fig. 2:**
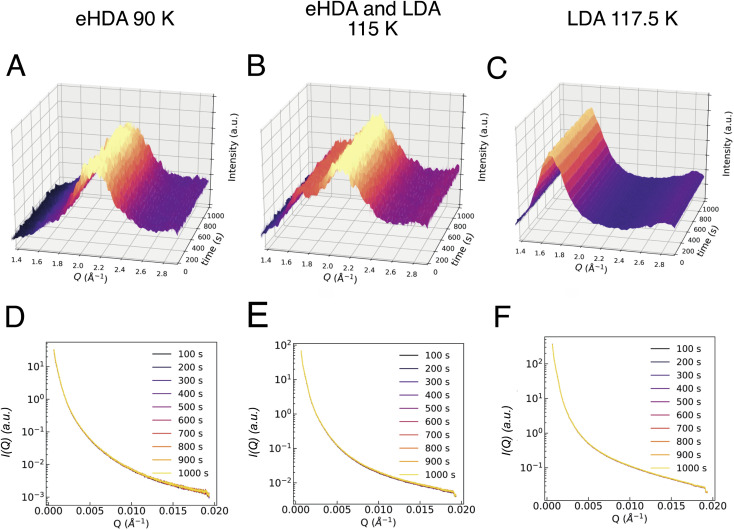
Azimuthally integrated intensity in the WAXS geometry over 1000 s of (A) eHDA at 90 K, (B) eHDA transforming to LDA at 115 K, and (C) LDA at 117.5 K. The azimuthally integrated intensities in the USAXS geometry of the same samples are shown below (D, E and F, respectively). Each line corresponds to the average of 100 s. Therefore, the plot labelled as 100 s is the average for the first 100 s, the one labelled as 200 s is the average from 101 to 200 s, *etc.* Temperatures as measured at cold finger, T-sample might be slightly higher.

**Fig. 3:**
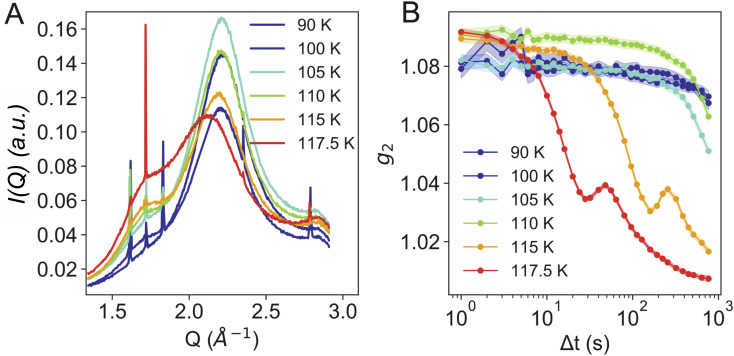
A) Azimuthally integrated intensity in WAXS geometry of eHDA at different temperatures during heating. (B) The corresponding intensity autocorrelation functions, *g*_2_,at different temperatures recorded in SAXS geometry at *Q* = 0.007 Å^−1^. Temperatures as measured at cold finger, T-sample might be slightly higher.

Additionally, the sample was monitored at larger scattering angles (WAXS) by a second detector to characterize the different states occurring throughout the measurement: HDA, LDA or crystalline ice. Fig. 2 shows the characteristic first diffraction maxima (WAXS) for three selected temperatures (A: 90 K, B: 115 K, C: 117.5 K) over the duration of 1000 s. We do not observe any structural changes within these 1000 s. At 115 K the double maximum reveals a coexistence of eHDA and LDA, without any significant changes during the measurement. We note that the sample stability during the 1000 s of the experiment is observed both in the WAXS and the USAXS region. Fig. 2D–F show the averaged (100 s) integrated intensity in the USAXS, plotted for the three selected temperatures. Once more, we see that the structure remains stable in this regime within the experimental time frame. Previous measurements at PAL-XFEL^23^ indicated that most sample compartments remained structurally stable at 115 K even after several hours. For the further XPCS analysis only stable sample spots (within the 1000 s measurement) were selected. In Fig. 3A we show how the eHDA sample transforms into LDA upon the stepwise heating. A heating rate of 5 K min^−1^ was used between each chosen temperature. Nevertheless, considering that the temperature was not changed for approximately 1 h between two subsequent temperature steps, the overall heating rate was of around 0.09 K min^−1^.

In Fig. 3A we can also observe contamination by hexagonal ice, seen by the characteristic Bragg reflections, especially between 1.5 Å^−1^ and 2 Å^−1^. We assume that the hexagonal ice originates from condensation during the sample transfer process. Condensation is also visible in some of the images in Fig. 1C as white “fluffy” ice sitting both on the copper grid and on the ice. In contrast, eHDA, once taken out of the piston-cylinder setup, looks relatively transparent while kept inside liquid nitrogen. We have carefully compared sample spots with and without previous condensation and cannot find an influence of the weakly bound hexagonal ice to the transition dynamics of HDA to LDA. The ice only causes additional Bragg-peaks which can be considered part of the background. We aimed to find clean sample spots throughout the experiment to reduce the background. Sometimes the “fluffy” ice even falls apart. Even if it would be in close contact to HDA, the crystallisation is kinetically hindered at the temperatures used, hence the contamination does not affect our results.

Fig. 3B shows the calculated g_2_ functions at different temperatures. It is evident that upon heating the dynamics become faster. At temperatures of 115 K and above, the dynamics are not only considerably faster, but display additional oscillations. We assume that the oscillatory behavior relates to the macroscopic expansion of the sample, as the HDA to LDA transition is accompanied by a 20% change in density. In previous measurements,^24^ powdered bulk samples had been confined between two diamond windows which did not allow for a fast expansion. This process requires further investigation and will be addressed in a separate study.

**Fig. 4:**
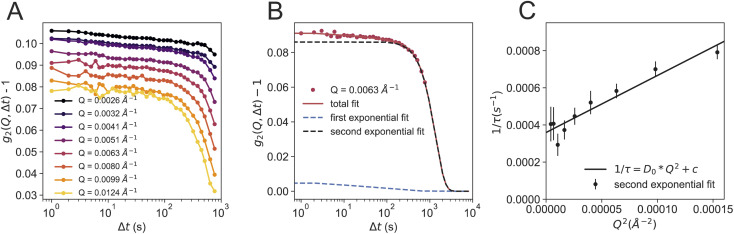
(A) Intensity autocorrelation functions *g*_2_ (*Q*, Δ*t*) calculated at different *Q* for eHDA at 110 K. (B) Example of experimental data fitted with a combination of two exponential functions: the blue dashed, black dashed and red solid lines indicate the first, second, and resulting double exponential fit, respectively. (C) The characteristic time obtained from the exponential fit plotted as 1/*τ* over *Q*^2^. The black solid line depicts the result of a fit *1*/*τ* = *D*_0_*Q*^2^ + *c* with *D*_0_ = 3.1 Å^2^ s^−1^ and *c* = 0.00036 s^−1^.

Fig. 4 shows further analysis of the dataset at 110 K. The g_2_ functions are calculated for different selected *Q*-regions over the whole area of the detector. The data can be fitted with a double exponential decay (Fig. 4B), as used in our previous study on powdered bulk samples.^24^ The main (second) relaxation process at 110 K does not show a clear *Q*^2^-dependence (Fig. 4C), as observed in our previous study.^24^ A *Q*^2^-dependence is only evident when adding an offset (*1*/*τ* ∼ *D*_0_*Q*^2^ + *c*), in which case the derived diffusion coefficient is *D*_0_ = 3.1 Å^2^ s^−1^. This diffusion coefficient is smaller than the one previously reported, which can be associated to a variety of factors: first of all, the sample geometry, thickness and morphology are completely different, as we have no windows and the sample is a thin sheet of ice. Secondly, using USAXS geometry allows to use a larger X-ray beam of 100 μm ×100 μm, which resulted in an X-ray dose two orders of magnitude lower than the previous study.

We additionally analysed the data by using a power law fit *Γ*(*Q*) = *Γ*_0_^*p*^ (Fig. S4A†) which is able to describe the data well, using an exponent of *p* = 0.7. We also observed that whilst we retain a compressed exponential behavior, *γ* decreases from around 2 to *ca.* 1.5 at higher *Q* (Fig. S4B†) indicating that at 110 K we have not yet entered a pure liquid state, as *γ* > 1. A *γ* = 2 was found in colloidal glasses for stress-dominated dynamics.^44^ Additionally, a cross over from Brownian motion (*γ* = 1) to ballistic motion (*γ* = 2) is observed around *T*g^45–47^ for silica tracer particles inside a glass forming matrix such as polypropylene glycol,^46^ related to a hyperdiffusive and strongly correlated particle motions inside the solvent.

**Fig. 5:**
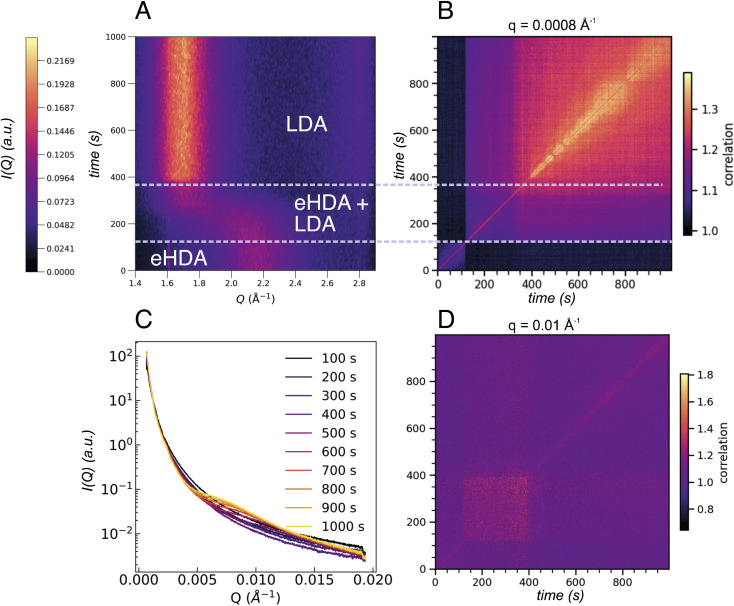
(A) Contour plot of the integrated intensity in the WAXS range of an eHDA sample heated to 117.5 K. The sample is transforming within the 1000 s of the measurement, allowing us to observe three regions: one where the eHDA is present, followed by the coexistence of eHDA and LDA, and the full transformation into LDA. (B) Two-time correlation plot at *Q* = 0.0008 Å^−1^ of the same sample where the three distinct regions are highlighted which show a transition from faster dynamics to slower dynamics once the transition has finished. (C) Integrated intensity in the USAXS range of the eHDA sample at 117.5 K where each line corresponds to the average of 100 frames equivalent to 100 s. (D) Two-time correlation plot at a *Q*-value of 0.01 Å^−1^ which falls in the region where a broad feature develops in the SAXS regime.

We note that not all sample spots throughout the Cu-grid behave exactly the same way, because of small variations in sample thickness, thermal contact or, eventually, the sample history. Fig. 1C also shows that not all grid-holes are perfectly filled. The ice sheets can break apart and remain as a partially filled hole for several hours at temperatures below 115 K, as monitored by the WAXS signal.^23^ Above 115 K, some sample spots can fully transform within seconds as observed in Fig. 5A showing the *I*(*Q*) as a function of experimental time for a sample at 117.5 K. This out-of-equilibrium-dynamics can best be visualized using the corresponding two-time correlation function (TTCF) (Fig. 5B). TTCFs are used to understand the time-dependence of dynamics, for example during the aging of gels and glasses or the change of dynamics during heating.^34,48–51^ The TTCF function is defined as:4
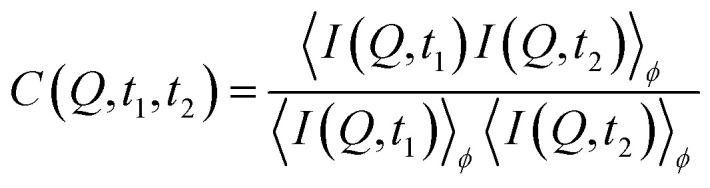
where *I*(*Q*, *t*) is the scattered intensity at a specific *Q*-value at time *t*, and 〈…〉_*ϕ*_ represents the average over pixels corresponding to the wave vectors |*Q*| = *Q* ± Δ*Q* in the azimuthal angle range of *ϕ* ± Δ*ϕ*. In this specific case, we averaged over the whole angular range of the detector. The width of the diagonal contour in the 2D-plots is scaling with the characteristic time *τ*, larger widths thus indicate slower dynamics (larger *τ*) and *vice versa*.^50,51^ In other words, the 2D-plots in Fig. 5 and S3† can be interpreted as such, that a narrow intensity on the diagonal line indicates rather fast dynamics, while a broad and smeared out intensity marks “slower” dynamics, as the correlations perpendicular to the diagonal line decay more slowly.

In Fig. 5B three distinct dynamical regions are visible, indicated by the dashed lines, which can be directly connected to the structural changes observed in Fig. 5A. Structurally we can discriminate between HDA, the transformation between 120 s and 370 s where both HDA and LDA coexist, and finally pure LDA. The three regimes show different dynamics at different *Q*-values as can be observed in Fig. S3.† Additionally, the USAXS integrated *I*(*Q*) (Fig. 5C) now shows a clear change throughout the 1000 s of the experiment, especially in the range between 0.005 Å^−1^ and 0.015 Å^−1^. The underlying dynamics of eHDA appear to be similar at different *Q* ranges (compare first dynamic region in Fig. S3†): we can see that while the sample is still in the eHDA state, the width of the diagonal contour is the smallest, a change in the dynamics then occurs during the phase transition starting at around 120 s. For very low *Q*-values (as plotted in Fig. 5B), the width of the diagonal contour is narrow, hence the transition from the high-to low-density regime is fast and liquid-like at the given length scale of around (2π/0.008) nm = 785 nm. Due to the fast transition, the system is still in a non-equilibrated state. Moreover, the limited time frame of around 200 s does not allow to extract the diffusion coefficient of the potential liquid state. In the third regime, we observe that the width is significantly broader and increases over time, indicating a slowing down when the glassy LDA state relaxes over time. This could be related to heat dissipation after the exothermic phase transition^40^ when the sample enters the low-density glassy state (LDA), which is expected to have slower dynamics than its liquid counterpart. At large scattering angles (Fig. 5D), however, the dynamics behave differently, rather slow dynamics are observed during the transition with faster dynamics once pure LDA is formed. The length scale can be estimated by (2π/0.1) nm = 62 nm. At this length scale (*Q* = 0.01 Å^−1^) a change in the integrated SAXS signal is observed (Fig. 5C). In summary, the dynamics in amorphous ices behave different at different length scales. A crossover can be observed in Fig. S3.†

### 3.2 Crystallisation of LDA

**Fig. 6:**
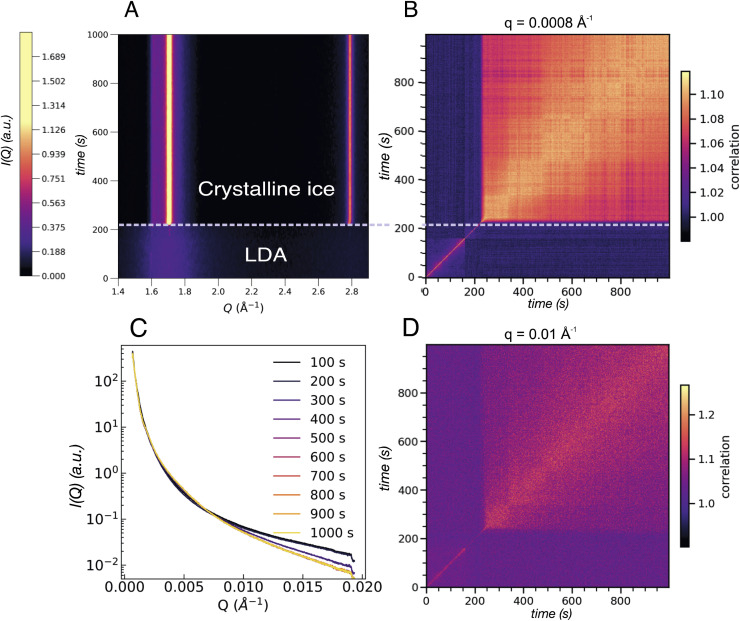
(A) Contour plot of the integrated intensity in the WAXS geometry of an LDA sample heated to 120 K. Two regions can be distinguished: one where the LDA is present followed by crystallisation occurring after *ca.* 200 s. (B) Two-time correlation plot at 0.0008 Å^−1^ of the same sample where the two regions are evident as an abrupt change from faster to slower dynamics once crystallisation has occurred. (C) Integrated intensity in the USAXS range of the LDA sample at 120 K where each line corresponds to the average of 100 frames equivalent to 100 s. (D) Two-time correlation plot at *Q* = 0.01 Å^−1^ which falls in the region where a broad feature develops in the SAXS regime in the eHDA sample (see Fig. 5C). The two distinct regions are present once more with the same slowing down in the dynamics as in B.

After the full transformation to LDA, we studied the crystallization dynamics by first quenching the LDA from 120 K to 80 K, followed by further heating in a stepwise manner. At moderate and fast heating rates, crystallization is usually observed at 160 K, as also displayed in Fig. 1. In Fig. 6 we show another sample spot where the transition into crystalline ice occurs already at 120 K, within the 1000 s of the measurement. Similar to the example of an early transition in eHDA, we assume that the chosen sample spot transforms earlier due to sample history, a thinner sample or a less efficient thermal contact at this particular spot. This example therefore does not represent an equilibrated LDA sample.

Fig. 6A shows the integrated intensity over time where abrupt crystallization at 205 s is evident. The two prominent lines at around 1.7 Å^−1^ and 2.8 Å^−1^ indicate the formation of stacking disordered ice *I*_sd_ with high cubicity.^41^ The corresponding XPCS analysis shows two regimes of dynamics for all length scales. Based on the width of the diagonal contour we can see that the dynamics strongly slow down directly after crystallisation, as expected. Two selected *Q*-values are shown in Fig. 6B for *Q* = 0.0008 Å^−1^ and *Q* = 0.01 Å^−1^ in Fig. 6D. The overall change of *I*(*Q*) is shown in Fig. 6C.

## 4. Conclusions

We studied the transition from high- to low-density amorphous ice, and from LDA to crystalline ice by probing self-standing thin layers of ice within a copper grid (Fig. 1A). We were able to probe the structure of the ice within each grid hole, due to the small X-ray beam sizes of 19 μm × 32 μm (XFEL) and 100 μm ×100 μm (storage ring), for the XFEL dataset even on a single shot basis. We followed the structural transition from HDA to LDA at different heating rates, namely 10 K min^−1^ using XFEL pulses, and a slow and stepwise heating of approximately 0.09 K min^−1^ where spontaneous ultrafast transition was observed for selected sample spots. At fast heating rates the transition takes place at approximately 130 K. In agreement with previous X-ray measurements using powdered samples, the transition takes place in an abrupt and distinct manner. In contrast, when using slow heating rates the transition occurs more gradually, as previously observed.^29,41^

XPCS studies in USAXS geometry allowed us to investigate the dynamics of amorphous ices at nm-length scales. In agreement with our earlier XPCS studies on powdered bulk samples,^24^ we observe an acceleration of the dynamics with increasing temperature. Our derived diffusion coefficient at 110 K of 3.1 Å^2^ s^−1^ is one order of magnitude smaller than in our previous study at similar temperature,^24^ however, the current study did not evaluate the dynamics at higher temperature. There are different possible causes for the observed smaller diffusion coefficient associated to the differences between the samples used in Perakis *et al.*^24^ These are mainly related to the sample geometry (free-standing thin ice layer *vs.* a 10 times larger powder sample between windows) and to our current setup which used a larger X-ray beam and lower X-ray dose. Our previous XPCS measurements on bulk samples^24^ as well as previous calorimetric and dielectric studies^40^ found the glass transition of eHDA to be at around 110 K. The exponential decay at 110 K can be fitted with a compressed exponential behavior with *γ* > 1.5. We propose two different scenarios to explain these observations: First, *γ* > 1.5 can be related to a hyperdiffusive behavior around the postulated *T*_g_ of eHDA at around 110 K, caused by nm-sized patches of water molecules that start a correlated motion. Second, the *γ*-value could be explained by stress-relaxation inside the glassy matrix. The presence of these motions can be the origin of the offset in the *Q*^2^-dependence. Lastly, we observe an additional dynamical component above 115 K, causing an oscillatory behavior in the temporal correlation function g_2_ which has not been previously reported.^24^ The underlying difference and nature of the additional motion at higher temperatures needs to be investigated further and will be addressed in a separate work.

Our studies have demonstrated that amorphous ice can be prepared as a free-standing ice-sheet inside a vacuum environment. Nevertheless, not all sample holes remain intact and some spots may exhibit different thermal history due to either an unevenly distributed thermal contact or mechanical stress. By choosing small enough probes, like micron-sized focused X-rays, thermally stable spots can be selected easily. By probing the out-of-equilibrium spots we can study the non-equilibrium dynamics within the 1000 s duration of our XPCS experiment. We have demonstrated that the HDA-LDA transition shows different dynamics at different length scales. In contrast, upon crystallization of the sample, we observed that the same dynamical behavior is present at all different *Q*s, *i.e.* a uniformly slowing down of the dynamics upon crystallization at all length scales. The ice then forms a stacking disordered ice *I*_sd_ with high cubicity.^42,43^ This finding might have an impact on understanding crystallization from amorphous ice in summer-mesospheric clouds,^8^ as also the μm-sized ice layer resembles a more realistic model system.

## Author contributions

Following CRediT contributor roles, authors contributed as follows. Conceptualization & Supervision: K. A.-W., F. P., G. G., A. N., F. L.; investigation (experiments): M. L.-P., H. L., A. K., K. H. K., M. R., F. D., N. S., F. W, K. A.-W.; resources & software (X-ray part): F. P., K. H. K., M. R., F. D., M. S., F. W., F. L.; resources (sample preparation): M. L.-P., H. L., A. K.; data analysis and visualization: M. L.-P., H. L.; writing original draft: K. A.-W., M. L.-P., H. L., F. L.; all authors have edited the draft and given approval to the final version of the manuscript.

## Conflicts of interest

The authors declare no competing financial interest.

## Supplementary Material

EA-002-D2EA00052K-s001
