# Supplementary material for: Using coherent X-rays to follow dynamics in amorphous ices
Source: Environ Sci Atmos. 2022 Sep 13;2(6):1314–23. doi: 10.1039/d2ea00052k (PMC9648632; doi:10.1039/d2ea00052k)
Supplement: EA-002-D2EA00052K-s001 [file EA-002-D2EA00052K-s001.pdf]

Electronic Supplementary Material (ESI) for Environmental Science: Atmospheres.
This journal is © The Royal Society of Chemistry 2022

Supporting Information:

Using Coherent X-rays to Follow Dynamics in Amorphous Ices

Marjorie Ladd-Parada[1], Hailong Li[1,2], Aigerim Karina[1], Kyung Hwan Kim[3], Fivos Perakis[1], Mario Reiser[1], Francesco Dallari[4], Nele Striker[4], Michael Sprung[4], Fabian Westermeier[4], Gerhard Grübel[4,5], Anders Nilsson[1], Felix Lehmkühler[4,5], Katrin Amann-Winkel[1,2,6,*]

1. Department of Physics, Stockholm University, Roslagstullsbacken 21, 10691 Stockholm, Sweden
2. Max-Planck-Institute for Polymer Research, Ackermannweg 10, 55128 Mainz, Germany
3. Department of Chemistry, POSTECH, Pohang 37673, Republic of Korea
4. Deutsches Elektronen-Synchrotron DESY, Notkestr. 85, 22607 Hamburg, Germany
5. Hamburg Centre for Ultrafast Imaging, Luruper Chaussee 149, 22761 Hamburg, Germany
6. Institute of Physics, Johannes Gutenberg University Mainz, Staudingerweg 7, 55128 Mainz, Germany

Unravelling the fundamental properties of water and water´s phase diagram is highly relevant for our understanding of water. Our studies give insight to the structural and dynamical properties of different amorphous ices and their crystallization. X-ray photon correlation spectroscopy (XPCS) was used to study the dynamics of high-density amorphous (HDA) ice upon heating. We follow the structural transition from HDA to low-density amorphous (LDA) ice, by using wide-angle X-ray scattering (WAXS), for different heating rates. Performing the XPCS study at ultra-small angle (USAXS) geometry allows us to characterize the transition dynamics at length scales ranging from 60 nm - 800 nm. This supporting information gives detailed information about our investigation on possible beam induced heating, prior to the experiment, as well as further comparison of two different samples and a detailed overview supporting Figure 5 of the main manuscript.

Absorber dependence

We tested different numbers of absorbers to avoid X-ray induced dynamics (Figure S1) and beam induced heating of the sample. Based on the estimated relaxation times (Figure S1B), we determined that the dynamics using either 12 or 16 absorbers are similar. We selected then the use of 16 absorbers, which refers to the number of Si foils (each foil with a thickness of 25 μm) and results in a final X-ray flux of 6.85×10^8 photons/s at an area of 100 μm x100 μm.

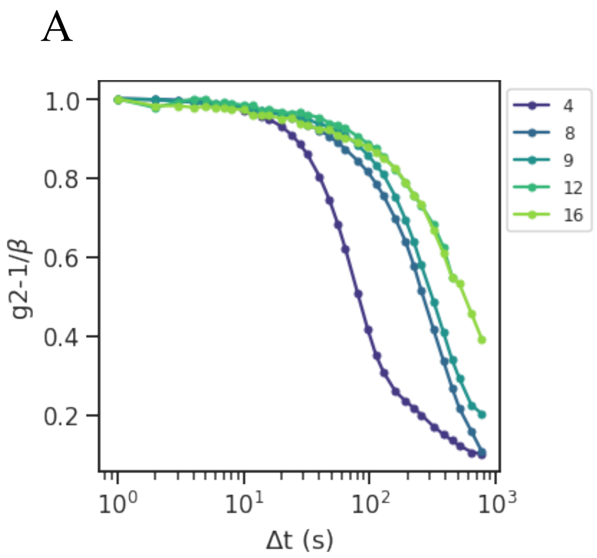

Figure S1. A. Absorber dependence of the temporal correlation functions at Q= 0.007 Å and B. relaxation times as a function of the number of absorbers (Si-foils of 25 μm each).

Difference in thermal stability amongst samples

Contrasting previous measurements on powdered bulk samples of ice, we here prepared the sample as a free-standing thin ice-layer inside a 0.1 mm thick Cu-disk with 1.5 mm hole-structure (see main text for details). X-ray measurements have been done in vacuum using a cryostat. We used two equally prepared eHDA samples for studying the eHDA to LDA transition. However, due to differences in sample thickness, or heat transfer and varying thermal contact, their thermal stability can differ. For example, Sample A (Figure S2 A to C) which is shown in the text, fully transforms when 120 K are reached, whilst Sample B (Figure S2 D to F) still retains an eHDA fraction at 122.5 K. Nevertheless, in both cases we can observe that there is an acceleration of the dynamics upon heating, and the development of oscillations at higher temperatures.

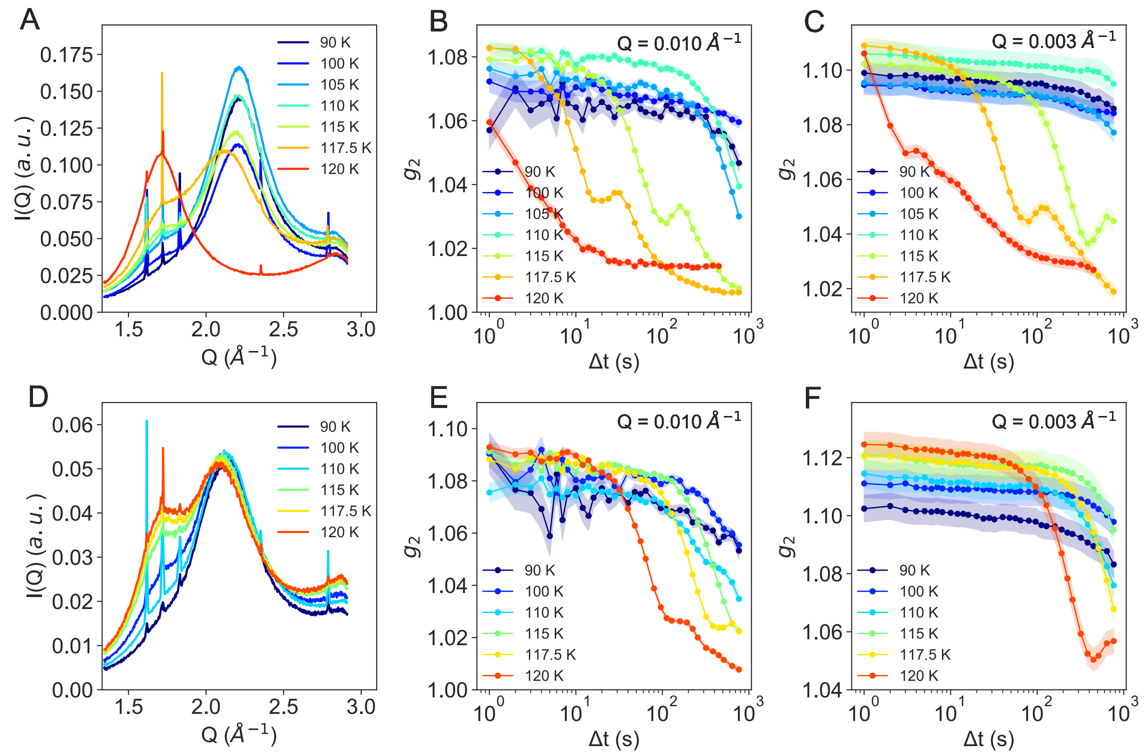

Figure S2. A) WAXS I(Q) scattering patterns of sample A at different temperatures. B) and C) Time correlation functions of sample A at different temperatures and Q-values. D) WAXS I(Q) scattering patterns of sample B at selected temperatures. E) and F) Time correlation functions of sample B at different temperatures and Q-values.

Two-time correlation functions at different Q-values

As mentioned in the main manuscript, structural changes are observed in the USAXS region during the eHDA to LDA transition at 117.5 K. They have different dynamical behaviours at different Q-values as shown by the two-time correlation functions.

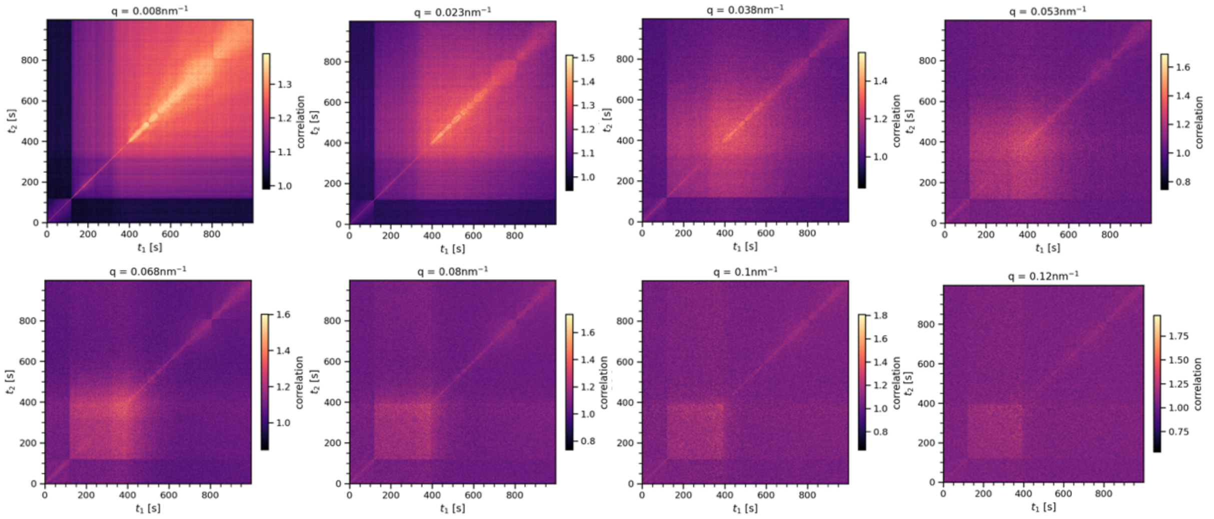

Figure S3. Two-time correlation functions of eHDA transforming to LDA at 117.5 K at 8 different Q-values.

Power law fittings of tau and gamma values

According to the shape of g_2 functions presented in Figure 4A we fitted the g_2 curves with a double exponential decay function (Figure 4B):

$$g_2(Q, \Delta t) = 1 + A \cdot \exp\{-2[\Gamma_1(Q)\Delta t]^{\gamma_1}\} + (\beta - A) \cdot \exp\{-2[\Gamma_2(Q)\Delta t]^{\gamma_2}\} \quad \text{(S1)}$$

where A is the amplitude bound to the interval $[0, \beta]$. The second and third terms on the right side of equation S1 represents the fast and slow decays, respectively. $\Gamma_1 = {}^{1}/_{\tau_1}$ and $\Gamma_2 = {}^{1}/_{\tau_2}$ are the fast and slow relaxation rate, and γ_1 and γ_2 are the corresponding KWW exponentials.

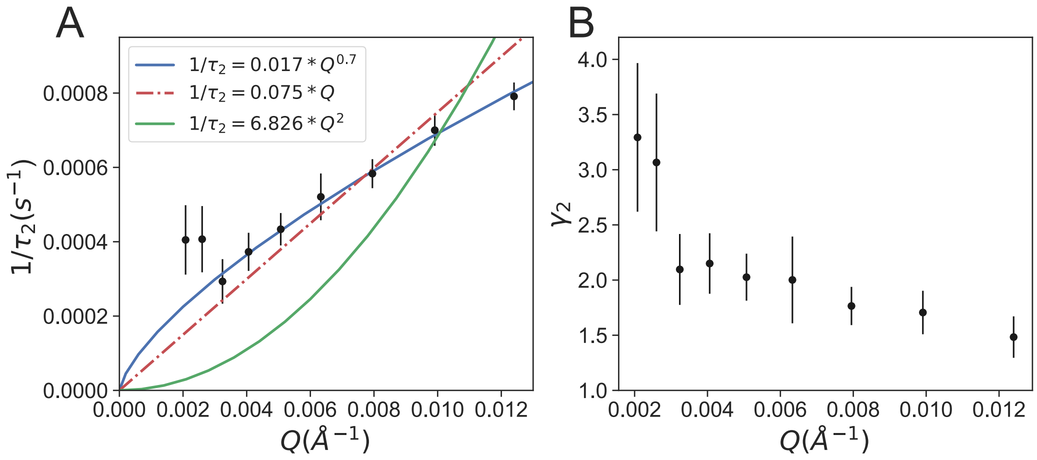

Figure S4. A) The characteristic time τ_2 extracted from the second exponential fit of g_2 curves presented in Figure 4A is plotted as $1/\tau_2$ as a function of Q. The blue, red and green lines are fittings to a power law $\Gamma(Q) = \Gamma_0 * Q^p$. B) The KWW exponent γ_2 of the second exponential fit as a function of Q.
